# An Efficient Plant Regeneration and Transformation System of Ma Bamboo (*Dendrocalamus latiflorus* Munro) Started from Young Shoot as Explant

**DOI:** 10.3389/fpls.2017.01298

**Published:** 2017-07-27

**Authors:** Shanwen Ye, Changyang Cai, Huibo Ren, Wenjia Wang, Mengqi Xiang, Xiaoshan Tang, Caiping Zhu, Tengfei Yin, Li Zhang, Qiang Zhu

**Affiliations:** Basic Forestry and Proteomics Center, Fujian Provincial Key Laboratory of Haixia Applied Plant Systems Biology, Haixia Institute of Science and Technology, College of Forestry, Fujian Agriculture and Forestry University Fuzhou, China

**Keywords:** regeneration, transformation, Ma bamboo, shoots

## Abstract

Genetic engineering technology has been successfully used in many plant species, but is limited in woody plants, especially in bamboos. Ma bamboo (*Dendrocalamus latiflorus* Munro) is one of the most important bamboo species in Asia, and its genetic improvement was largely restricted by the lack of an efficient regeneration and transformation method. Here we reported a plantlet regeneration and *Agrobacterium*-mediated transformation protocol by using Ma bamboo young shoots as explants. Under our optimized conditions, embryogenic calluses were successfully induced from the excised young shoots on callus induction medium and rapidly grew on callus multiplication medium. Shoots and roots were regenerated on shoot induction medium and root induction medium, respectively, with high efficiency. An *Agrobacterium*-mediated genetic transformation protocol of Ma bamboo was established, verified by PCR and GUS staining. Furthermore, the maize *Lc* gene under the control of the ubiquitin promoter was successfully introduced into Ma bamboo genome and generated an anthocyanin over-accumulation phenotype. Our methods established here will facilitate the basic research as well as genetic breeding of this important bamboo species.

**Key achievements**: A stable and high efficiency regeneration and *Agrobacterium*-mediated transformation protocol for Ma bamboo from vegetative organ is established.

## Introduction

Bamboo species belong to the grasses family (*poaceae*) and are well-known for their great economic, social, and cultural value (Buckingham et al., 2014). They cover tropical, subtropical, and temperate zones of the world and are considered as the prime renewable resource for biomass production due to their short rotation period and fast-growing culm (Tanaka et al., 2003). Ma bamboo (*Dendrocalamus latiflorus* Munro) is one of the most important semitropical clumping bamboo species and forms abundant forests in Asia (Buckingham et al., 2014). It produces nutritious bamboo sprout and reaches up to 20 m in height and 25–30 cm in diameter. Its products are widely used in construction, handicrafts, paper pulping, and as efficient agents for conservation of water and soil (Scurlock et al., 2000; Qiao et al., 2013). The demands for economically important bamboo species like Ma bamboo are on the rise due to its great benefits to human beings (Singh et al., 2013).

Many bamboo species have quite long and irregular flowering habit, therefore it is nearly impossible to introduce favorable traits through conventional breeding. Genetic engineering provides a powerful tool in plant breeding programs, while genetic manipulation largely depends on the availability of efficient *in vitro* regeneration systems, which became one of the major bottlenecks in bamboo research. Until now, the successful regeneration protocols were limited to few bamboo species. Regeneration of bamboo plantlets was reported for the first time from the zygotic embryo of *Bambusa arundinacea* (Mehta et al., 1982). Since then several bamboo species were regenerated successfully from mature seeds of *D. latiflorus, D. strictus, Bambusa multiplex*, and *D. hamiltonii* (Rao et al., 1985; Yeh and Chang, 1987; Yuan et al., 2013)*;* or anther of *B. oldhami, D. latiflorus*, and *B. beecheyana* var. *beecheyana* (Yeh and Chang, 1986a,b; Tsay et al., 1990; Woods et al., 1992; Qiao et al., 2013). The anthers and mature embryos from seeds were the most frequently used explant resources in bamboo tissue culture, whereas there are several disadvantages by using these starting materials. Firstly, it is not so easy to get bamboo anther or seeds at anytime and anywhere as the flowering time of bamboo is long and erratic. Secondly, it has the risk of generating tissues or calluses comprising a chimera of haploid, diploid, tetraploid, and dodecaploid cells (Qiao et al., 2013). In contrast, vegetative tissues such as young shoots are available at all times and are therefore ideal alternative explants for callus induction and plantlet regeneration. However, it is much more difficult to establish the regeneration platform using vegetative tissues as explant (Singh et al., 2013). To our knowledge, only one report showed successful plantlet regeneration from calluses derived from shoot tips of *D. hamiltonii* very recently (Zang et al., 2016), but unfortunately this protocol is species-specific, and could not be applied to other bamboo species.

In recent years, great progress has been made on Ma bamboo research at the genomic or transcriptomic level. For example, 10,345 ESTs library had been generated from young leaves of Ma bamboo (Gao et al., 2011), the complete nucleotide sequences of its chloroplast genome was also published (Wu et al., 2009) and transcriptomic resources are also available (Liu et al., 2012). By using next generation high-throughput sequencing technology and bioinformatics analysis, 84 conserved miRNAs have been identified in Ma bamboo (Zhao et al., 2013). Those data provide important gene resources for the future bamboo genetic improving. However, the development of bamboo transgenic technology has lagged behind, which largely restricted bamboo genetic breeding. The only successful callus induction and plant regeneration system from the anther of Ma bamboo was reported by Qiao et al. (2013). The same research group successfully transformed a bacterial *CodA* gene and produced the cold-tolerant transgenic Ma bamboo using anther-derived callus (Qiao et al., 2014). As we discussed above, plantlet regeneration from anther has several disadvantages for bamboo breeding, and cannot be widely used. To solve this problem, it is necessary to develop a regeneration and transformation protocol with vegetative tissues as the explants.

In the present study, we provided an efficient and reproducible protocol for Ma bamboo regeneration and *Agrobacterium*-mediated transformation. We successfully induced calluses starting from young shoots as explants and regenerated bamboo plantlet by optimizing tissue culture media. Based on this, we further established the *Agrobacterium*-mediated transformation protocol, and heterologously expressed the maize leaf color (*Lc*) gene in Ma Bamboo. The methodology reported here allows Ma bamboo regeneration and transformation started from easily obtainable vegetative tissue, which is an important step toward the genetic manipulation of this bamboo species in the future.

## Materials and Methods

### Plant Materials

Ma bamboo (*Dendrocalamus latiflorus* Munro) from Fujian Province of China was used in this study. The newly emerged shoots were collected from the 2 to 3 years’ old plants propagated through cuttings and vigorously grown in the greenhouse of Fujian Agriculture and Forestry University at a temperature of 26 ± 2°C and 16 h light/8 h dark photoperiod, with the light intensity of 100 μmol/m^2^/s and the humidity of 60–65%. For surface disinfection, young shoots were cut into small pieces, and then incubated with commercial antiseptic agents for 60 min, followed by washing under tap water for 2 h. The materials were soaked in 75% ethanol for 1 min, and then washed with sterilized water 3–5 times under aseptic conditions. Then they were sterilized with 0.1% bichloride of mercury for 8 min followed by 5–7 times washing with the sterilized water. After washing, the young shoots were cut into 0.5–1 cm length with nodule at the end and then were used for callus induction. Calluses were induced and multiplied in the dark, whereas shoots and roots were induced in the light with the light intensity of 60–70 μmol/m^2^/s and the photoperiod of 16 h light/8 h dark. The whole regeneration procedure was performed at a temperature of 26 ± 2°C.

### Callus Induction and Subculture

Based on previous reports on bamboo regeneration, we designed various combinations of auxins [including 4 mg/L 2,4-dichlorophenoxyacetic acid (2,4-D), 2 mg/L NAA, and 2 mg/L IBA] and cytokinins [1 mg/L kinetin (KT) and 2 mg/L BAP] for callus induction. Other compositions of callus induction medium (CIM) include basic MS salts supplied with 30 g/L sucrose and 4.2 g/L phytogel. The pH of the medium was adjusted to 5.8. The shoots were transferred to the same CIM for subculture every 15–20 days. The callus gradually appeared 1.5 months after induction, and the decayed tissues were carefully removed. And the calluses were transferred into the new fresh medium for subculture and the following callus multiplication. After approximately 3 month subculture on CIM, they were transferred to the callus multiplication medium (CMM) which contains 3/4 MS basal medium supplemented with 30 g/L sucrose, 3 g/L sorbitol, 250 mg/L PVP, 2 mg/L 2,4-D, and 4.2 g/L phytogel for callus multiplication. The yellow and compact calluses were transferred onto the fresh CMM medium every month. This step took around 7–8 months. The healthy, creamy-yellow and compact calluses produced in this step were selected for shoot induction and *Agrobacterium*-mediated transformation.

### Adventitious Shoot Induction from Callus

The calluses multiplied on the CMM were transferred onto shoot induction medium (SIM) to induce shoots in the light. We investigated the effects of various concentration of auxins (0–1 mg/L NAA, 0.5–1 mg/l IAA) and cytokinins [2 and 6 mg/L BAP, 0.01 and 0.1 mg/L thidiazuron (TDZ), 0.5 and 1 mg/L zeatin (ZT)] on inducting adventitious shoots. Other compositions of SIM were full-strength MS medium, 30 g/L sucrose, 4.2 g/L phytogel, and the pH value was adjusted to 5.8. Shoots were induced in a 90-mm diameter petri dish with around 30 ml medium; each petri dish contains around 30 calluses. The calluses were transferred on the same fresh medium for subculture every month.

### Rooting of the Shoots and Transplantation

When the shoots are 2–3 cm high and form a cluster containing 2 or more shoots, the clusters were transferred onto root induction medium (RIM), of which the composition include: 1/2 MS medium, 30 g/L sucrose, 8 g/L agar, and various kinds of auxin. Here we mainly investigated the effect of three kinds of auxin (NAA, IBA, and IAA) on inducing root. When the length of inducted roots reached 3–5 cm, the regenerated plantlets were transferred into the potting soil and grown in the greenhouse.

### Optimization of the Concentration of Kanamycin and Hygromycin for the Selection of Putative Transgenic Lines

The calluses were transferred to CMM or SIM medium supplemented with 0–50 mg/L hygromycin or 0–100 mg/L kanamycin. The survival of calluses and the frequency of regenerated shoots and roots were statistically analyzed after 2 months. All those experiments had at least three independent replicates, each of which comprised around 100 samples.

### Binary Vector Construction

The whole coding sequence of the maize *Lc* gene (GeneBank accession No. M26227.1) was amplified using P35s-Lc plasmid as the template (Wang et al., 2016). The product was first recombined into entry vector *PDNOR 207* by a BP Gateway reaction (Invitrogen, China), and subsequently cloned into the modified pCambia1301 binary vector harboring the Gateway cassette sequence and the maize Ubiquitin (Ubi) promoter by a LR Gateway reaction (Invitrogen, China). The primers used in this experiment were: Forward: 5′-GGGGACAAGTTTGTACAAAAAAGCAGGCTTAATGGCGCTTTCAGCTTCCCG-3′ and Reverse: 5′-GGGGACCACTTTGTACAAGAAAGCTGGGTATCACCGCTTCCCTATAGCTT-3′. The sequencing-confirmed plasmid was introduced into *Agrobacterium* for further usage.

### Transformation of Ma Bamboo

A single colony of *Agrobacterium tumefaciens* containing the binary vector was inoculated overnight in 3 ml YEB medium (5 g/L tryptone, 1 g/L yeast extract, 5 g/L nutrient broth, 5 g/L sucrose, and 0.5 g/L MgSO_4_⋅7H_2_O) supplemented with 50 mg/L kanamycin and 25 mg/L rifampicin on a shaker at 200 rpm at 28°C in dark. A 2-ml aliquot was diluted to 200 ml YEB medium containing 50 mg/L kanamycin and 200 μmol/L acetosyringone (AS), and we grew this bacteria suspension until it reached an OD_600_ of 0.6–0.8 on a shaker at 100 rpm. Agrobacteria cells were collected by centrifugation at 5000 rpm for 5 min, and the pellet was re-suspended in AAM liquid medium (Hiei and Komari, 2008) supplemented with 200 μmol/L AS, and then the density of bacteria was adjusted to reach an OD_600_ of 0.8. The selected yellow-compact and healthy calluses were submerged in the bacteria cell suspension and put under vacuum (-0.8 bar) for 15 min, and then gently shook for 15 min at 26°C. Excess bacteria were removed by drying on the sterilized filer papers. The calluses were placed on the NB basal medium (Hiei and Komari, 2008) containing 200 μM AS and 2 mg/L 2,4-D for co-cultivation at 25°C in dark for 4 days. After that, the calluses were washed four times with the sterilized water containing 500 mg/L carbenicillin and then were dried on the sterilized filter paper. The calluses were placed on CMM medium containing 500 mg/L carbenicillin and 35 mg/L hygromycin for *Agrobacterium* inhibition and hygromycin-resistant callus selection. The concentration of carbenicillin was gradually reduced to 200 mg/L for the following subculture. After 4 months’ selection on CMM medium, the yellow and compact hygromycin-resistant calluses were transferred to SIM medium supplemented with 35 mg/L hygromycin and 200 mg/L carbenicillin for shoot induction for another 2 months. The regenerated hygromycin-resistant shoots were used for root induction on RIM medium.

### Optimization of the Factors Influencing Transformation Efficiency

Several factors that potentially affect the transformation efficiency were optimized; here we mainly focused on three factors: Agrobacterium strains (LBA4404, GV3101, and EHA105), the density for the bacterial cell (OD 0.2–1.0), and the duration for Agrobacteria-callus co-cultivation (0–6 days). The frequencies of regenerated hygromycin-resistant shoots were used to assess the efficiency of transformation. The procedure was performed as described above, with the variation of these three parameters for optimization. At least 100 calluses were tested with three independent replicates.

### Molecular Analysis of the Transgenic Plants

Genomic DNA was isolated from the leaves of putative transgenic and control plants using the DNA extraction kit (Qiagen, China) as described by the manufacturer. The presence of the 35S promoter and *HPTII* gene was confirmed by PCR analysis using primers specific for 35S promoter (Forward: 5′-TGCCATCATTGCGATAAAGGAAAG-3′) and *HPTII* gene (Reverse: 5′-CTGCTGCTCCATACAAGCCAACC-3′). *GUS* gene was detected using primers specific for the 35S promoter (Forward: 5′-TGCCATCATTGCGATAAAGGAAAG-3′) and *GUS* gene (Reverse: 5′-GAGCGTCGCAGAACATTACA-3′). The PCR reactions were carried out using the Premix Taq DNA Polymerase Kit (Clontech, China) as described by the manufacturer. The expected size of 35S-HPTII product was 1044 bp under our PCR conditions: initial denaturation at 95°C for 1 min; 40 cycles at 98°C for 10 s, 59°C for 30 s, and 72°C for 58 s; and a final extension step at 72°C for 10 min. For 35S-GUS fragments, the expected fragments of 1866 bp were obtained with the PCR conditions: initial denaturation at 95°C for 1 min; 40 cycles at 98°C for 10 s, 60°C for 30 s, and 72°C for 90 s; and a final extension step at 72°C for 10 min.

For maize *Lc* gene expression analysis, total RNA was extracted from young shoots of putative transgenic and untransformed seedlings using Qiagen RNeasy Plant Mini Kit (Qiagen, China), and the reverse transcription reactions were performed by using reverse transcriptase ReverTraAce (Clontech, China) according to the manufacturer’s instruction. qRT-PCR was performed using SYBR^®^ Premix Ex Taq^TM^ II (Clontech, China). Specific primers for *Lc* gene were designed using primer premier 5 software (qLC-Forward: 5′-ACGGGAGCAGCACAGGAAAT-3′ and qLC-Reverse: 5′-CGACGCTTTGTTCACCCTGT-3′). The expression data were normalized against the expression of the previously reported reference genes specific primers: qAC-Forward: 5′-TGCTCTCCCCCATGCTATCCTTC-3′ and qAC-Reverse: 5′-ATGTCCCTCACAATTTCCCGCTC-3′ (Liu et al., 2014). Three independent biological replicates with four technical repeats were performed.

### GUS Staining

Histochemical GUS staining was performed as described previously (Jefferson et al., 1987). Briefly, hygromycin-resistant callus and regenerated shoots were immersed in a reagent containing 2 mM 5-bromo-4-chloro-3-indolyl-β-D-glucuronide (X-Gluc), 5 mM Ferro-Ferricyanide buffer, 0.1 M Na-phosphate buffer (pH = 7.0), 10 mM EDTA, and 0.1% Triton X-100, and incubated at 37°C for 24 h.

### Anthocyanin Measurement

Anthocyanin was measured as described previously (Neff and Chory, 1998). Briefly, young seedlings were extracted with acidified methanol with 0.1 M HCL (85:15, v/v) for 24 h at 4°C in darkness. The absorbance of the extracted solution was measured at 530 and 657 nm, and the content of anthocyanin was calculated using the formula: A_530nm_ - 0.25 × A_657nm_, and the data was expressed as anthocyanin per fresh weight. The experiments were performed at least three times independently.

### Statistical Analysis

All the experiments in this study were repeated at least three times, and ANOVA and the Student’s *t-*test were used for statistical analyses.

## Results

### Callus Induction from Stem

**FIGURE 1:**
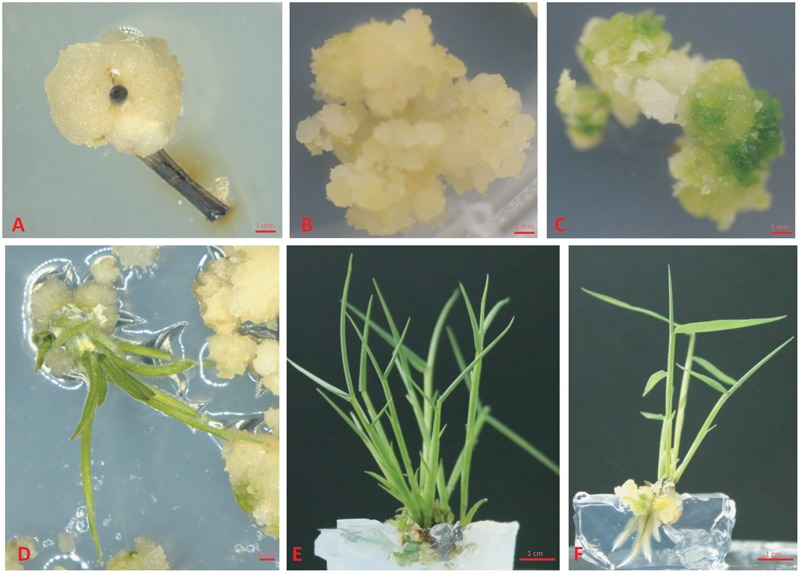
Regeneration of plantlet from young shoots of Ma bamboo. **(A)** Callus was induced from the shoots of Ma bamboo. **(B)** The callus grown on the callus multiplication medium. **(C)** Callus turns green on shoot induction medium. **(D)** The induced shoots on shoot induction medium. **(E)** The adventurous shoots grow in cluster. **(F)** Root was induced on root induction medium.

Previous reports showed that MS medium (Murashige and Skoog, 1962) is the most effective media for both direct and indirect organogenesis in bamboo tissue culture (Singh et al., 2013), therefore we chose MS medium for the whole regeneration procedure. As the first step for callus induction, the young shoots were sterilized and cut as we described in “Materials and Methods,” and then they were placed on CIM. Based on our initial test, 2,4-D is a key phytohormone for Ma bamboo callus induction (data not shown), we further optimized the concentration of 2,4-D, and tested the effect of its combination with cytokinins (IBA, KT, and TDZ) on inducing calluses, and we found that 2,4-D and IBA have strong effects on bamboo callus induction, while the combination of 2,4-D with cytokinines (KT and TDZ) significantly reduced the ratio of bamboo callus induction (**Table 1**). Of those combinations, the best medium for inducing Ma bamboo callus was: MS medium supplemented with 30 g/L sucrose, 8 mg/L 2,4-D, 0.5 mg/L IBA, and 4.2 g/L phytogel, and with this optimized medium, the rate of callus induction was around 53% after 2.5 months’ induction (**Table 1**). At around 1.5 months, three types of calli were developed from the explants grown on CIM medium: white and friable, yellow-compact and brownish (**Figure 1A** and **Supplementary Figure S1A**). By DAPI staining, we found the most cells from the white and friable callus do not contain nucleus (**Supplementary Figure S1B**), indicating they lost their ability to multiply. The calluses were transferred to CMM for multiplication approximately 3 months after induction. We found that 2,4-D was the key phytohormone that sustained embryogenic callus proliferation, and the strength of the MS medium also slightly affects the physiological status of the embryogenic calluses (data not shown). In summary, the compositions for the optimized CMM were: 3/4 MS basal medium supplemented with 30 g/L sucrose, 3 g/L sorbitol, 250 mg/L PVP, 4.2 g/L phytogel, and 2 mg/L 2,4-D. During the subculture of calluses in this medium, the friable non-embryogenic calluses were discarded by themselves, and at around 8 months after callus induction on CMM, the embryogenic calluses became yellow, compact and healthy (**Figure 1B**). The calluses produced in this step were used for the following shoot induction, and they were also the targeting materials for the genetic transformation.

**Table 1:** Effects of 2,4-dichlorophenoxyacetic acid (2,4-D) on compact callus induction from nodule of the young shoot.

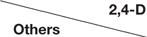	0	2 mg/L	4 mg/L	8 mg/L
N/A	–	31.83 ± 7.57	33.5 ± 6.80	36.67 ± 4.46
IBA (0.5 mg/L)	–	29.33 ± 6.50	37.83 ± 4.71	53.17 ± 4.07
KT (3 mg/L)	–	9.67 ± 3.14	12.67 ± 4.93	12.50 ± 9.22
TDZ (0.01 mg/L)	–	12.17 ± 3.37	19.17 ± 6.08	21.83 ± 6.62

*Values represent the means ± standard errors. At least three independent experiments with 100 explants for each repeat were performed. The data was collected 2.5 months after induction.*

### Shoot Induction

It is known that cytokinin plays a major role in stimulating shoot formation in *in vitro* plant tissue cultures, and high cytokinin over auxin ratio promotes shoot formation from callus (Skoog and Miller, 1957). Various kinds of cytokinin such as 6-BA, KT, and ZT have been frequently used in bamboo tissue culture (Singh et al., 2013). Thidiazuron is a synthetic cytokinin-like substance, and it is highly effective for shoot regeneration in the tissue culture of woody plants (Maheshwari and Kovalchuk, 2011). In this study, various combination of cytokinins (6-BA, TDZ or KT) and auxins (NAA and IAA) were tested in different concentrations (For cytokinins: 2 mg/L and 6 mg/L BAP, 0.01 mg/L and 0.1 mg/L TDZ, 0.5 mg/L and 1 mg/L ZT, and for auxins 0.5 mg/L and 1 mg/L NAA) (**Table 2**). The frequency of shoot induction was around 50% on MS medium supplied with 30 g/L sucrose, 4.2 g/L phytogel, BAP 2 mg/L, and NAA 0.5 mg/L after 1.5-month shoot induction. Different phytohormone combinations including 0.1 mg/L TDZ alone or 0.1 mg/L TDZ + 0.5 mg/L NAA also effectively induced shoots, with frequencies of 50% (**Table 2**). Under the growth condition of our lab, the calluses started to turn green 2 weeks after they were put on the SIM medium containing BAP 2 mg/L + NAA 0.5 mg/L, and the regenerated shoots continued to grow another 1.5 months for root induction (**Figures 1C–E**).

**Table 2:** Effects of phytohormones on shoot induction.

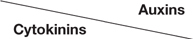	0	NAA 0.5 mg/L	NAA 1 mg/L	IAA 0.5 mg/L	IAA 1 mg/L
BAP 2 mg/L	22.2 ± 2.77	49.96 ± 2.35	19 ± 2.56	30.77 ± 4.54	20.27 ± 1.84
BAP 6 mg/L	13.5 ± 0.83	29.74 ± 6.45	14.92 ± 5.74	6.01 ± 2.46	35.7 ± 3.27
TDZ 0.01 mg/L	24.62 ± 1.36	46.61 ± 2.30	11.85 ± 2.31	17.32 ± 0.65	14.89 ± 3.94
TDZ 0.1 mg/L	49.47 ± 1.51	49.55 ± 12.49	14.26 ± 3.71	14.84 ± 3.62	7.23 ± 1.19
ZT 0.5 mg/L	14.25 ± 0.31	15.54 ± 5.84	44.03 ± 2.67	28.56 ± 2.88	5.39 ± 1.23
ZT 1 mg/L	6.81 ± 1.32	11.22 ± 2.22	23.98 ± 1.12	24.69 ± 2.38	28.81 ± 4.40

*Values represent the means ± standard errors. At least three independent experiments started with 100 calluses for each repeat were performed. The data was collected 1.5 months after induction.*

### Rooting of the Plantlets

Auxin is the most important factor determining formation of the adventurous root, and a high auxin to cytokinin ratio favors root production in plant tissue culture (Vanneste and Friml, 2009; Vanstraelen and Benkova, 2012). Mainly, three types of auxin were used for root induction: native IAA, synthetic NAA, and IBA, and different plant species respond quite differently to various kinds of auxin in inducing adventitious root formation (De Klerk et al., 1997). Shoot clusters with 2–4 buds were used for rooting. Our results showed that all these three kinds of auxin effectively induced root, while the best RIM medium was 1/2 MS basal medium supplemented with 1 mg/L IAA, with the root regeneration frequency of 72.8 ± 1.7% at around 1 month after induction (**Figure 1F** and **Table 3**). Under our growth conditions, the roots were regenerated within 1 month after being transferred on the RIM medium.

**Table 3:** Effects of phytohormones on root induction.

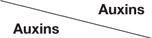	NAA 0.5 mg/L	IBA 0.5 mg/L	IAA 0.5 mg/L
NAA 0.5 mg/L	52.84 ± 0.66	39.98 ± 0.85	51.39 ± 1.87
IBA 0.5 mg/L	52.9 ± 1.92	44.32 ± 1.83	55.68 ± 1.83
IAA 0.5 mg/L	22.89 ± 1.62	65.76 ± 2.06	72.83 ± 1.66

*Values represent the means ± standard errors. At least three independent experiments started with 100 samples for each repeat were performed. The data was collected 1 month after induction.*

### Transplantation

**FIGURE 2:**
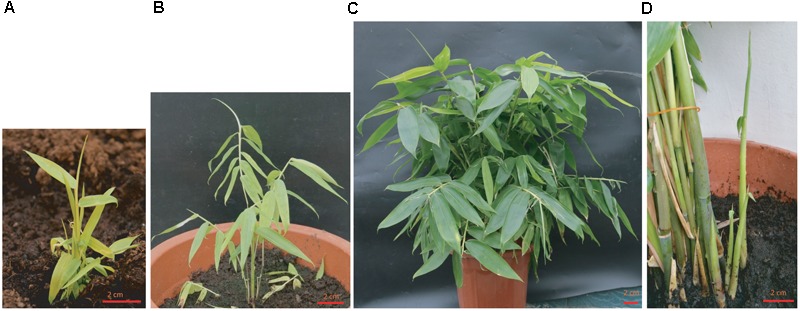
Growth status of regenerated plants after transplanted into soil. **(A)** The growth status of the regenerated plantlet 1-month after being transferred to the soil and grown in greenhouse. **(B)** The growth status of 3-month old plant. **(C)** One-year old plant grown in the green house. **(D)** Multiple new clusters of shoots were growing out of the soil. Bar = 2 cm.

After rooting, the plants were transferred into soil and grown in the greenhouse. Previous report showed that the anther-derived haploid plants were hard to be transplanted into soil and died due to a lack of vigor (Tsay et al., 1990). For vegetative organ-derived plant using our method, the survival rate was as high as 98% after transplantation. For the first 3 months, the above-ground parts of plants grew slowly, but the root grew quickly in the soil at this stage (data not shown). After around 3 months, the shoot started to grow vigorously and rapidly, with many new bamboo culms emerged from the plant, and the plants reached 50–90 cm within 1 year (**Figure 2**).

### Optimization of the Concentration of Antibiotic for Transformation Selection

Our initial test showed that bamboo calluses were insensitive to kanamycin (data not shown), whereas they were highly sensitive to the elevated concentrations of hygromycin (**Supplementary Figure S2**). With increased concentration of hygromycin, the survival ratio of callus and the frequency of bamboo shoot induction were significantly impaired. Based on our dose-dependent test, CMM and SIM containing 35 mg/L hygromycin were used for screening the putative transgenic plants (**Supplementary Figures S2A,B**). For root organogenesis, our results showed that hygromycin at 30 mg/L completely abolish the calluses’ ability to generate roots (**Supplementary Figure S2C**). However, practically we do not use hygromycin selection at the root induction step to have the maximum survival ratio and the better growth performance of the hygromycin-resistant shoots. Carbenicillin (Cb) were widely used to inhibit the growth of *Agrobacterium* after incubation (Cheng et al., 1998). Our results showed that the morphology and the frequency of the regenerated shoots remain unchanged in response to as high as 500 mg/L Cb (data not shown), practically we use 400 mg/L Cb for the inhibition of *Agrobacterium* at the beginning, and then reduced to 200 mg/L in the following subculture.

### Setup of the Transformation Procedure

**FIGURE 3:**
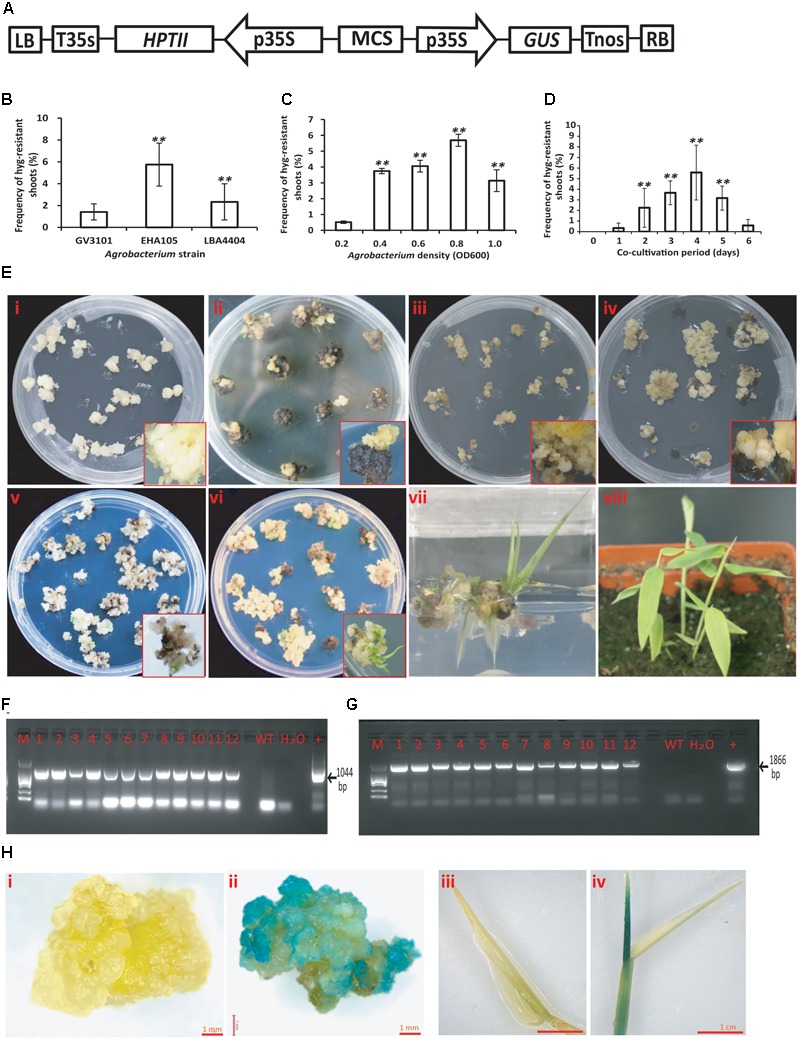
*Agrobacterium*-mediated transformation of Ma bamboo. **(A)** Schematic representation of key elements of T-DNA in the binary vector pCAMBIA1301. LB, left border; T35S, terminator of CaMV35S; HPTII, hygromycin phosphotransferase gene; p35S, promoter of CaMV35S; MCS, multiple cloning sites; GUS, uiA β-glucuronidase gene; Tnos, NOS terminator; RB, right border. **(B–D)** Various factors that affect the frequencies of hygromycin-resistant shoot induction of Ma bamboo; including *Agrobacterium* strain **B**, the density of *Agrobacterium* **C**, and the duration of co-cultivation **D**. Data were collected around 7 months after infection. Results were presented as means and standard errors from at least three independent experiments. At least 100 calluses were used for each experiment. ^∗∗^ indicates significant differences in comparison to control at *p* < 0.01 (Student’s *t*-test). **(E)** Key steps for *Agrobacterium*-mediated transformation of bamboo callus. **(i)** Callus status before *Agrobacterium* infection. **(ii)** New calli emerged from the transformed callus grown on the hygromycin containing medium. **(iii)** Morphology of 2-month old hygromycin-resistant calli. **(iv)** Status of the hygromycin-resistant calli that were used for shoot induction. **(v)** Green callus on shoot induction medium containing hygromycin. **(vi)** Regenerated shoots from hygromycin-resistant callus. **(vii)** The induced root on root induction medium. **(viii)** Regenerated plantlets grown in the soil. The enlarged pictures of represented callus were shown inside the panels. **(F)** PCR verification of the putative transgenic lines using 35S-forward and *HPTII-*reverse primers, which resulted in a 1044 bp product. **(G)** PCR verification of the putative transgenic lines using 35S-forward and *GUS*-reverse primers, which produced 1866 bp fragment. **(H)** GUS staining of the putative transformed callus **(ii)** and shoot **(iv)**, with the uninfected callus or shoots **(i,iii)** as the negative control.

Around 8-month old calluses grown on CMM were co-cultivated with the *Agrobacterium* containing the pCambia 1301 binary vector for transformation (**Figure 3A**). The *A. tumefaciens* strain is one of the major factors that affect the transformation efficiency. Our results showed that EHA105-infected calluses produced the highest hygromycin-resistant shoots while GV3101 showed much lower virulence (**Figure 3B**). Therefore, EHA105 was chosen for the following examination of other factors that may potentially affect transformation efficiency, such as the optical density of bacterial suspension (OD) and the period for co-cultivation. We found that infection of calluses using EHA105 with a bacterial suspension of OD 0.8 and then co-cultivating the callus and *Agrobacterium* for 4 days at 25°C generated the best results based on the number of hygromycin-resistant shoots (**Figures 3C,D**). Vigorously grown calluses were selected for infection (**Figure 3E-i**). They were transferred to CMM containing hygromycin after co-cultivation and rapidly became brown. But after further 1.5 months’ selection, small and yellow calli started to grow from their mother calluses (**Figure 3E-ii**). Of the 298 co-cultivated calluses, 204 produced hygromycin-resistant calli, with the transformation efficiency of 68.5%. Each calli was carefully transferred to the fresh CMM medium containing hygromycin for further growth (**Figure 3E-iii**). Some embryogenic embryos were developed around 3–4 months after infection (**Figure 3E-iv**). Such embryos were used for shoot induction on SIM containing 35 mg/L hygromycin. Green calluses appeared around 1.5 months after the transfer (**Figure 3E-v**), and continued to grow and develop shoots after another 1–2 month growth (**Figure 3E-vi**). The regenerated shoots were transferred to RIM without hygromycin for root induction (**Figure 3E-vii**). The regenerated plants were transferred to the soil and continued to grow in the greenhouse (**Figure 3E-viii**).

To verify the presence of the T-DNA in the genome of hygromycin-resistant shoots, genomic DNA from leaves of the putative transgenic plants was extracted and examined using PCR method. The expected 35S-HPTII and 35S-GUS fragments were detected (**Figures 3F,G**), indicating the presence of introduced T-DNA in the genome of the lines we tested. The hygromycin-resistant calluses and shoots were further analyzed by histochemical GUS staining analysis, and the results showed that the positive blue staining signals were detected in the hygromycin-resistant calluses and shoots (**Figures 3H-ii,iv**), whereas the GUS signals were not present in the control panels (**Figures 3H-i,iii**). In total, of the 298 calluses used for infection, 17 independent hygromycin-resistant plants were generated (5.7%), and 12 plants showed PCR positive, with a transformation efficiency of around 4%.

### Heterologous Overexpression of the Maize *Lc* Gene in Ma Bamboo Induces Anthocyanin Accumulation

**FIGURE 4:**
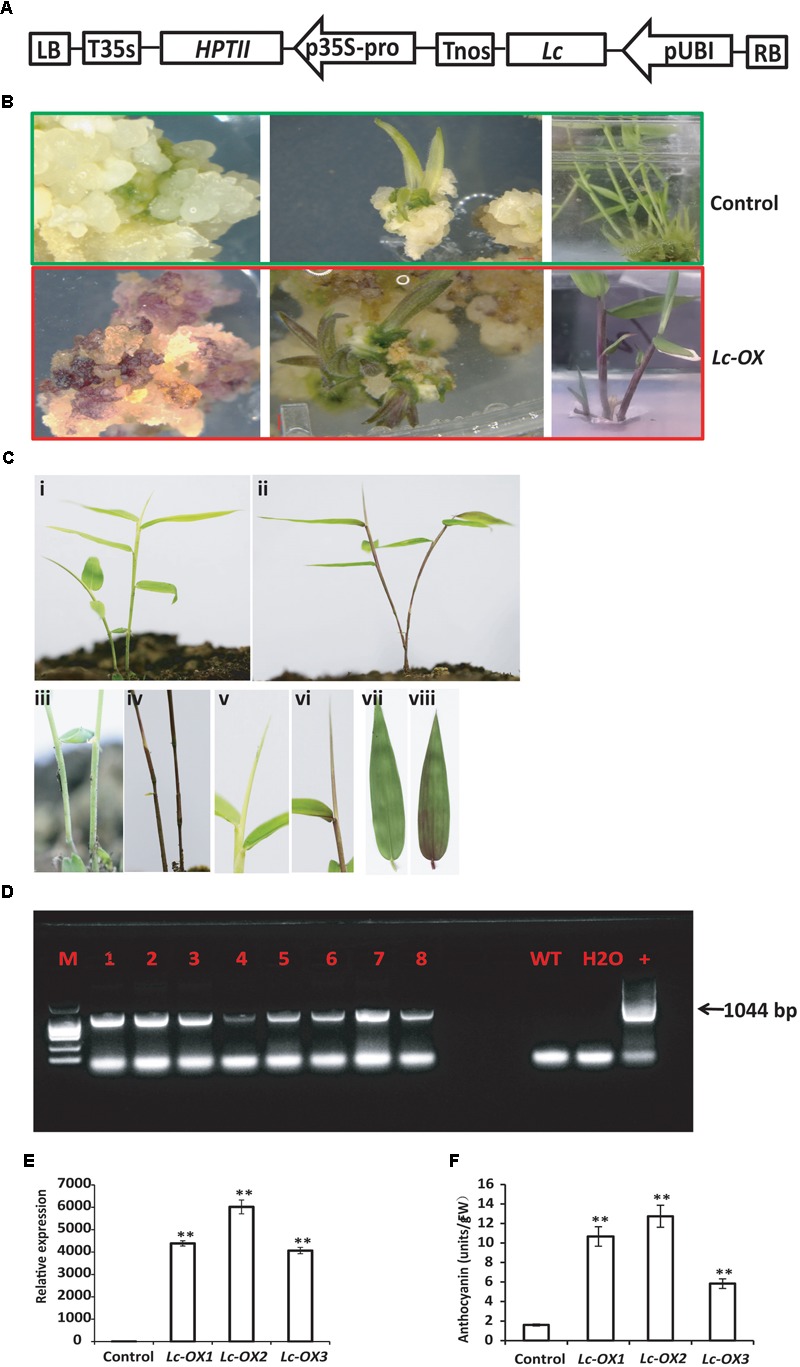
Overexpression of the maize *Lc* gene in Ma bamboo leads to the increased accumulation of anthocyanin. **(A)** Schematic representation of the putative inserted T-DNA region. The expression of maize *Lc* gene was controlled by ubiquitin promoter (pUBI), and *HPTII* gene driven by 35S promoter confers plant with hygromycin resistance. **(B)** Phenotypes of *Lc*-gene heterogonous expression lines during tissue culture. Purple pigment accumulation in the callus (left), regenerated shoot (middle and right panels) in the transgenic line (lower panel) compared with control (up panel). **(C)** Phenotype of *Lc*-gene heterogonous expression lines grown in the soil. Represented transgenic lines **(ii)** exhibit purple color compared with control **(i)**. The enlarged picture of stem **(iv)**, leaf blade **(vi)**, and leaf **(viii)** and their corresponding controls (**i,iii,v,vii**, respectively) were shown. **(D)** PCR analysis of the transgenic lines. The expected 1044 bp product was detected with genomic DNA from putative transgenic bamboo as template. **(E)** qRT-PCR analysis of *Lc*-gene heterogonous expression lines. Total RNA was extracted from control and three represented transgenic lines (*Lc-ox1, Lc-ox2, and Lc-ox3*), and the qRT-PCR was performed using *Lc* gene specific primers. The Actin gene of Ma bamboo was used as internal control. The data shown represent mean values and standard errors obtained from at least two independent experiments with four technical repeats. ^∗∗^ indicates significant differences in comparison to control at *p* < 0.01 (Student’s *t*-test). **(F)** Quantitative measurement of anthocyanins in 1-month-old seedlings of the control and three represented *Lc*-gene heterogonous expression lines. The data was shown as means and SD of three replicates. ^∗∗^ indicates significant differences in comparison to control at *p* < 0.01 (Student’s *t*-test).

Heterologous expression of the maize *Leaf color* (*Lc*) transcription factor which belongs to the basic helix-loop-helix (bHLH) family and regulates flavonoid pathway genes increases the anthocyanin pigments in creeping bentgrass, cotton, rice, and sweet potato (Han et al., 2009; Li et al., 2013; Fan et al., 2016; Wang et al., 2016), suggesting its potential usage as a visible marker for plant genetic transformation. To further confirm the validity of our bamboo transformation method and to check if maize *Lc* gene also functions in Ma bamboo, we constructed the binary vector that expressed the *Lc* gene under the control of Ubi promoter (**Figure 4A**), and transformed bamboo as we described above. During tissue culture process, the hygromycin-resistant calluses showed an unusual purple color in light, which never appears in the control calluses (**Figure 4B**). The following young regenerated shoots also appear to be red, the leaf sheath and leaf blade also exhibit purple, while the control plants are still green (**Figure 4B**). The transgenic lines were transferred into soil and the purple color stayed in stems, young shoots, and leaves (**Figures 4C-ii,iv,vi,viii**) compared with control (**Figures 4C-i,iii,v,vii**).

To verify that the T-DNA genes were inserted into the Ma bamboo genome, we performed PCR analysis. In total, 29 of the 37 hygromycin-resistant shoots showed the bands of the expected size (1044 bp), and results from 8 of the represented lines were shown in **Figure 4D**. Correspondingly all those lines showed anthocyanin over-accumulation phenotype. To check the expression of *Lc* genes of the transgenic lines, we randomly selected three transgenic lines and performed the qRT-PCR analysis. And our results also confirmed the integration and expression of *Lc* gene in those lines (**Figure 4E**). These results are correlated with the data of anthocyanin measurements, which showed much higher accumulation of anthocyanin in the transgenic lines (**Figure 4F**).

## Discussion

Bamboo serves as one of the most important natural resources to our human beings, whereas probably it is the only major agronomic crop that is nearly impossible to make improvements by selective breeding. Genetic engineering can overcome the limitations of traditional breeding of bamboo, however, the lack of a stable, convenient and efficient bamboo transformation protocol became the main bottleneck. Although the successful regeneration and transformation of Ma bamboo through anther-derived calluses was reported (Qiao et al., 2013, 2014), its routine usage was seriously limited due to the difficulties to get bamboo anther as starting materials and the ploidy problems of anther-derived regenerated plants. Therefore, we aim to establish new protocols for regeneration and transformation of Ma bamboo by using vegetative organs as explants.

The optimized combinations of plant hormones and growth regulators are the prerequisite of success in plant tissue culture. To establish a regeneration protocol that can be used for transformation, we screened the suitable growth medium containing various auxins and cytokinins step by step and successfully established the system. 2,4-D was required for the callus induction in Ma bamboo as reported in other bamboo species (Singh et al., 2013), and the synergistic effect of the combination of 8 mg/L 2,4-D and 0.5 mg/L IBA was also found in this process (**Table 1**). For shoot regeneration, high cytokinin/low auxin combinations are needed. Based on our tests, 2 mg/L BAP + 0.5 mg/L NAA effectively stimulated the shoot regeneration (**Table 2**); TDZ is widely used in the propagation and regeneration of many bamboo species (Singh et al., 2013), and 0.1 mg/L TDZ effectively induce shoot differentiation of Ma bamboo, with the highest frequency around 50% (**Table 2**). The quality and quantity of the shoot induction effects from 2 mg/L BAP + 0.5 mg/L NAA and 0.1 mg/L TDZ were comparable, and in our lab we often used 2 mg/L BAP + 0.5 mg/L as this combination was more economical. For root regeneration, IAA was more effective than other kinds of auxin (**Table 3**). Compared with the anther-based regeneration system, our system is more convenient to use because we started with young shoot as the explant, which avoid the ploidy problems and is much easier to get. More importantly, the anther-derived haploid plants could not survive long in the soil (Tsay et al., 1990), while our regenerated plants from shoot-derived callus vigorously grew (**Figure 2**), which shows another important advantage of our technique.

The frequency of shoot organogenesis from calluses was around 50%, which was sufficient for the following genetic transformation. However, it took around 8 months to get the good and healthy calluses that can be used for regeneration and transformation, and another 7–8 months to finish the transformation procedure, which was quite time-consuming and uncertain. The insufficient availability of embryogenic tissues, the low efficiency of the regeneration ratio, and the time-consuming processes were the main limitations for genetic transformation of bamboo. In the future, further optimization to shorten the time for getting high quality calluses, and the establishment of an embryogenic culture like previously reported in coffea (Ribas et al., 2011) will largely shorten the time for whole transformation procedure.

Several other factors determine the successful production of transgenic plants, such as the virulence of *Agrobacterium*, and suitable antibiotic selection marker. Our results showed that the *Agrobacterium* strain EHA105 is more effective than LBA4404 and GV3101. We further optimized the density of *Agrobacterium* for infection and the duration for co-culture to increase the transformation efficiency. After transformation, we noticed that the regeneration ability of hygromycin-resistant calluses was largely reduced on SIM and many could not regenerate plants. This was another main obstacle in the bamboo transformation, as previously reported in maize (Komari and Kubo, 1999). Further optimization of the transformation procedure to get more healthy calluses that can be used for shoot regeneration is still needed to increase the bamboo transformation efficiency.

The GUS, anthocyanin, and luciferase reporter genes have been widely used in plant genetic transformation, with the GUS reporter is the most commonly used. However, the detection of GUS is destructive and indirect. Compared with GUS, anthocyanin accumulation is easily observed by naked eyes. A number of reports showed the availability of anthocyanin as the reporter gene. In this project, we chose both GUS and anthocyanin as the reporters, and we successfully expressed GUS gene and the maize *Lc* gene into the genome of Ma bamboo. Our results showed that overexpression of *Lc* gene leads to the over-accumulation of anthocyanin, reflecting the validity of our transformation procedure.

With the availability of the regeneration and transformation protocol for young shoots of Ma bamboo, it is now feasible to introduce genes with important functions, and it will be a useful tool for basic research and genetic breeding of this important bamboo species in the future.

## Author Contributions

QZ conceived the project, designed experiments, and wrote the manuscript; SY, CC, and HR did the plasmid constructions and statistical analysis; WW, MX, XT, CZ, TY, and LZ performed the tissue culture related experiments.

## Conflict of Interest Statement

The authors declare that the research was conducted in the absence of any commercial or financial relationships that could be construed as a potential conflict of interest.

## Supplementary Material

FIGURE S1 Morphology of the induced calluses from young shoots. **(A)** The morphology induced calluses from young shoots. Left panel, friable callus; middle panel, yellow and compact callus; right panel, brown callus. Pictures were taken around 1.5 months after induction on CIM. **(B)** DAPI staining of the friable callus (left panel), as well as yellow and compact callus (right panel). The morphology of the represented calluses was shown in the red rectangle. Arrows indicate the positions of nucleus.

FIGURE S2 The sensitivity of hygromycin during the regeneration of Ma bamboo. Calluses were grown on CMM medium **(A)**, SIM medium **(B)**, and RIM **(C)** medium containing various concentration of hygromycin as indicated in the charts. The survival ratio of callus, the frequency of shoot regeneration, and the frequency of shoot regeneration was calculated after 2 months. All the experiments were repeated at least three times with 100 samples for each repeat. Data represents the average of three independent experiments and SD.
