# Supplementary figures and images for: An Efficient Plant Regeneration and Transformation System of Ma Bamboo (Dendrocalamus latiflorus Munro) Started from Young Shoot as Explant
Source: Front Plant Sci. 2017 Jul 27;8:1298. doi: 10.3389/fpls.2017.01298 (PMC5529393; doi:10.3389/fpls.2017.01298)

Supplemental Figure 1

A

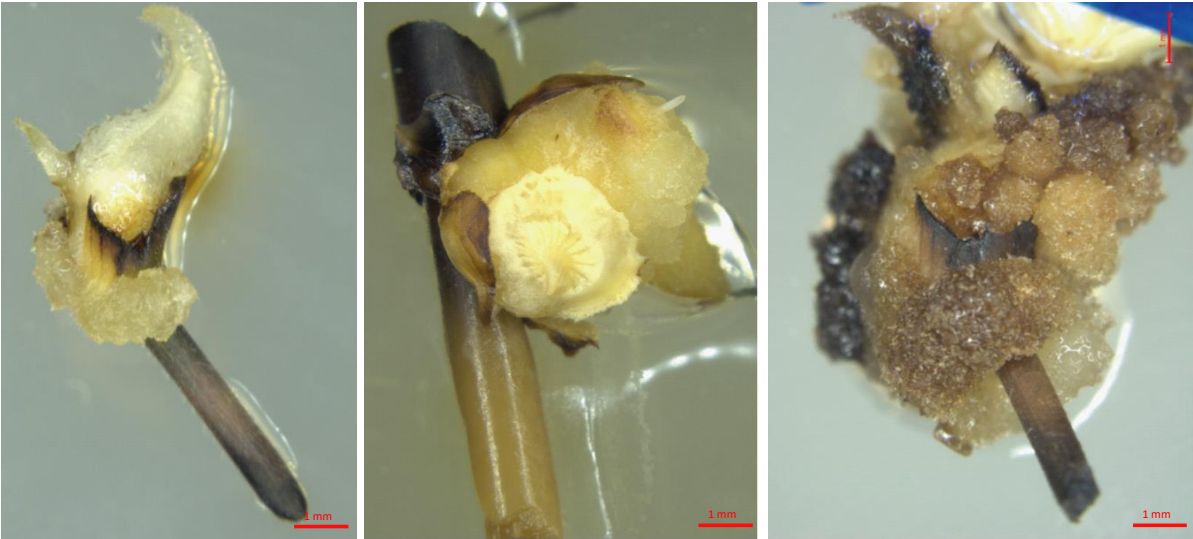

B

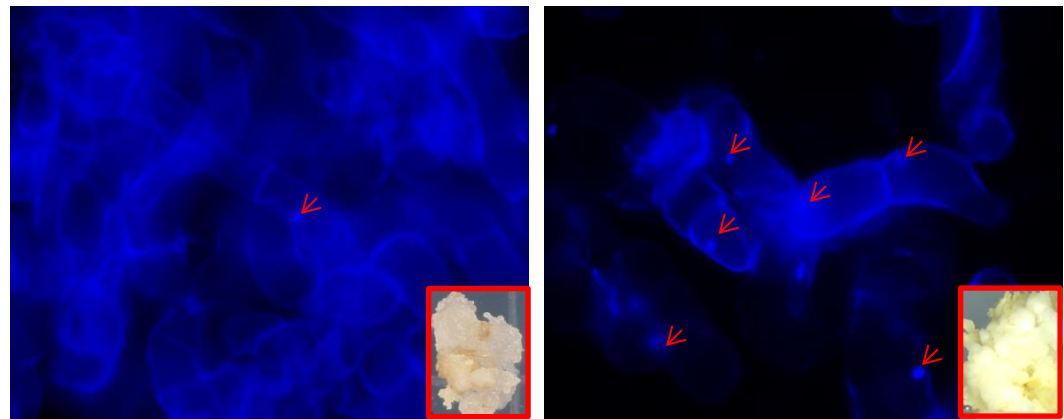

Supplemental Figure 2

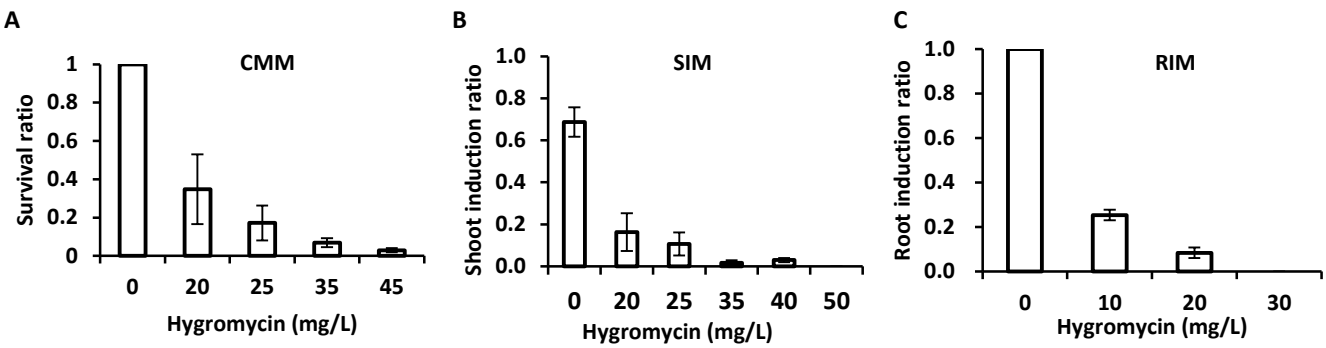

Supplement: FIGURE S1 — Morphology of the induced calluses from young shoots. (A) The morphology induced calluses from young shoots. Left panel, friable callus; middle panel, yellow and compact callus; right panel, brown callus. Pictures were taken around 1.5 months after induction on CIM. (B) DAPI staining of the friable callus (left panel), as well as yellow and compact callus (right panel). The morphology of the represented calluses was shown in the red rectangle. Arrows indicate the positions of nucleus. [file Presentation_1.pdf]
